# Hydrophobic Membrane Wettability: Effects of Salinity and Temperature

**DOI:** 10.3390/membranes15020058

**Published:** 2025-02-09

**Authors:** Orhan Kaya

**Affiliations:** Department of Mechanical Engineering and Mechanics, Lehigh University, Bethlehem, PA 18015, USA; ork216@lehigh.edu

**Keywords:** hydrophobic surface, wettability, temperature effects, salinity effects

## Abstract

In this study, molecular dynamics (MD) simulations were used to investigate the effects of salinity (NaCl) and temperature (25 °C and 80 °C) on the wettability of droplets on a realistically modeled hydrophobic PTFE (polytetrafluoroethylene) surface. Droplet sizes of 20, 25, and 30 nm were analyzed using both pure water and 8.45% NaCl solutions. The results indicated that salinity increased the contact angles, strengthening the PTFE’s hydrophobicity by disrupting the water’s hydrogen bonding at the interface and reducing the spreading area. Higher temperatures also led to an increase in contact angles by decreasing water structuring, although this effect was less pronounced than that of salinity. Ion concentration analysis revealed that a significant number of ions migrated away from the PTFE surface, a phenomenon further clarified through radial distribution function (RDF) analysis.

## 1. Introduction

Wettability, the study of liquid–solid surface interactions, is crucial in surface science [1,2], impacting fields like materials science, chemistry, and fluid dynamics [3,4,5]. It is primarily assessed by measuring the contact angle (θ_CA_), where the liquid–vapor interface meets the solid surface. Contact angles can be measured using computational [6] or experimental [7] methods and are categorized as superhydrophilic (θ_CA_ ≈ 0°), hydrophilic (0 < θ_CA_ < 65°), hydrophobic (65 ≤ θ_CA_ < 150°), superhydrophobic (θ_CA_ ≥ 150°), each indicating varying degrees of liquid affinity or repellency. To date, few studies have investigated how temperature and salt concentration affect water droplet wettability on solid surfaces. In 2006, Sghaier et al. [8] showed experimentally that water droplet contact angles on hydrophilic glass surfaces increase with sodium chloride (NaCl) concentration, due to ion adsorption. They noted that the exact mechanisms behind this need further study. In 2009, Jae Hyun et al. [9] used molecular dynamics (MD) simulations to study temperature effects on the contact angle of a pure water nanodroplet (~4800 water molecules) on a hydrophilic titanium dioxide (TiO_2_) surface. They found that increasing temperature decreased the contact angle, making the surface more hydrophilic due to reduced surface tension and hydrogen bonding. The authors suggested extending such studies to other surfaces for broader insights. In 2011, Christopher et al. [10] used MD simulations to study the contact angle of nanodroplets (~2000 water molecules) with NaCl and magnesium chloride (MgCl_2_) on a graphite-like surface. By adjusting Lennard–Jones parameters, they varied the surface’s hydrophilicity. They found that the addition of salts increased the contact angle, especially on more hydrophilic surfaces, but did not analyze the mechanisms behind this change. In 2015, Jun Zhang et al. [11] studied the wetting and evaporation of salt-water nanodroplets (~5800 water molecules) on hydrophilic platinum surfaces. They found that higher salt concentration increased the contact angle due to surface tension changes and suggested studying rough surfaces for further insights. In 2020, Xin Li et al. [12] found that increasing NaCl concentration in nanodroplets (~8400 water molecules) on a hydrophilic magnesium oxide (MgO(001)) surface led to more hydrophobic behavior due to ion hydration affecting water molecule ordering. They recommended studying temperature effects and rough surfaces for broader applicability. In 2022, Yijie Xiang et al. [13] experimentally showed that the contact angle of pure water droplets on hydrophobic polytetrafluoroethylene (PTFE) increased as the temperature rose from 25 °C to 80 °C. The droplet size effect was overlooked in the cited papers; however, Axel Verduzco et al. (2023) [14] found that the contact angle of water nanodroplets on graphite decreased from 70° to 63° as size increased from 2000 to 8000 molecules, with negligible effects beyond 8000. This factor could influence the results, but convergence with different droplet sizes was not tested in the cited papers. Moreover, previous studies primarily focused on hydrophilic surfaces (e.g., glass, titanium dioxide, magnesium oxide, platinum), leaving the impact of salinity and temperature on hydrophobic surfaces largely unexplored. Given PTFE’s widespread use in membrane technology, coatings, and biomedical applications, understanding wettability behavior in saline and high temperature environments is crucial. Therefore, this research investigated the effects of salinity and temperature on nanodroplet wettability on a realistically modeled rough hydrophobic PTFE surface using MD simulations, ensuring convergence by considering droplet sizes of 20, 25, and 30 nm. The selection of nanometer-scale droplet sizes in this study is justified by their relevance to real-world applications. The interaction of nanodroplets with membrane surfaces significantly impacts separation efficiency and selectivity, influencing phenomena such as fouling and permeability [15]. Studying droplet behavior at the nanometer scale also provides insights for designing microfluidic systems [16]. The mechanisms behind contact angle changes were investigated through ion concentration and density profiles, with the radial distribution functions (RDFs) quantifying NaCl distribution and its effects on interfacial properties and contact angles.

## 2. Methods

MD simulations using LAMMPS [17] were conducted to study the contact angle of NaCl-water nanodroplets on a PTFE surface. Post-processing of MD data was performed using OVITO [18] and VMD [19]. The Lennard–Jones parameters [20] and partial charges for PTFE, water [21], ions and simmulation settings [22,23] are provided in the Supplementary Materials.

### 2.1. Constructing the Simulation Model

**Figure 1:**
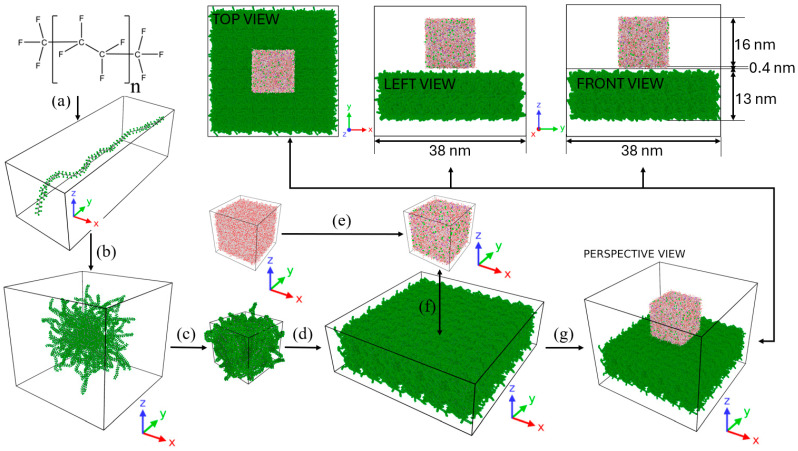
Simulation workflow: (**a**) Initial PTFE chain setup. (**b**) Replication, energy minimization, and 5 ns MD simulation at 298.15 K and 101.325 kPa. (**c**,**d**) Enlarged PTFE lattice. (**e**) Addition of Na^+^ and Cl^−^ to SPC/E water. (**f**) Merging equilibrated saline/pure water lattices with PTFE at 298.15 K and 353.15 K. (**g**) 3D and 2D lattice visualizations.

The amorphous PTFE model construction is shown in Figure 1. The PTFE chain, with CF_3_-(C_2_F_4_)_n_-CF_3_ repeating units, was built using force field data [24] from Orhan Kaya et al. [25], with ’n’ set to 50. Trifluoromethyl (CF_3_) groups cap both ends, as depicted in Figure 1a. The initial polymer chain was built using Avogadro [26] and duplicated to create 50 identical chains, which were positioned in a cubic lattice to realistically mimic hydrophobic PTFE. Energy minimization removed atomic overlaps, followed by a 5 ns NPT MD simulation to compress the box, in Figure 1b, and reach the experimental density of 2180 kg/m^3^ [27]. The cubic PTFE assembly was then replicated in Figure 1c six times along the x- and y-axes and twice along the z-axis to enlarge the system. The replicated model was then equilibrated for 1 ns using NVT MD simulations at 298.15 K and 353.15 K to prepare for merging with pure and saline water lattices. Initial cubic lattices of 16, 20, and 24 nm edge lengths were constructed to model pure and saline water using SPC/E water molecules. A minimum edge length of 16 nm was chosen based on Giovambattista et al. [28], who noted minimal line tension effects for edges larger than 3.25 nm. Packmol [29] placed water molecules with 2.0 Å spacing and a 1.5 Å safety margin to prevent boundary artifacts. Water molecules were deleted and Na^+^ and Cl^−^ ions inserted, as shown in Figure 1d, to achieve the target NaCl mass concentration (C_NaCl_) using Equation (1):

(1) $$C_{NaCl\;}=\frac{m_{Na^{+}}+m_{Cl^{-}}}{m_{\mathrm{water}}+m_{Na^{+}}+m_{Cl^{-}}}\times 100\%$$

Here, C_NaCl_ denotes the solution’s mass concentration (%), m_Na+_ and m_Cl−_ are the masses of sodium and chloride ions, and m_water_ is the water mass. Table 1 lists the water molecule counts, added Na^+^ and Cl^−^ ions, and C_NaCl_ for each of the three cubic lattices.

Six cubic configurations (three saline and three pure) were generated and equilibrated for 1 ns at 298.15 K and 353.15 K to ensure stability before merging with an amorphous PTFE model (Figure 1e).

These 12 configurations were positioned 0.4 nm above the highest PTFE atom and centered. The 12 nm edge-length droplet was merged and shown in Figure 1f from different views. The systems were then prepared for the equilibration process.

### 2.2. Equilibration Methodology and Contact Angle Measurements

**Figure 2:**
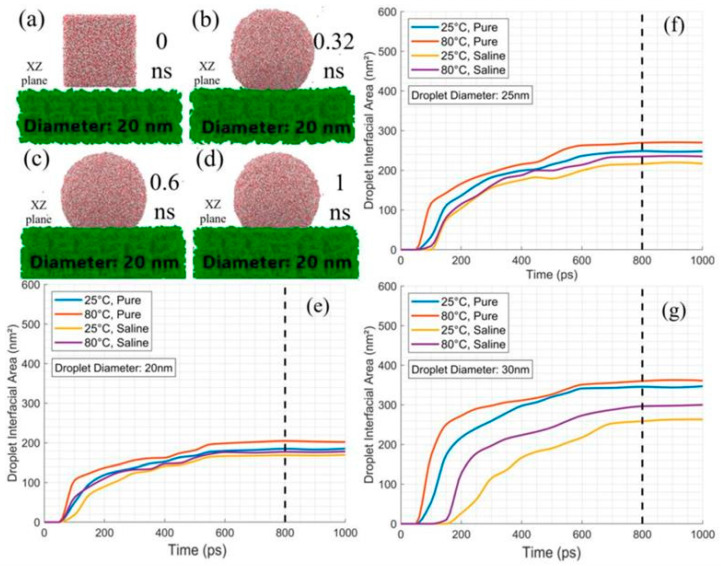
MD snapshots (**a**–**d**) show the equilibration of a 20 nm pure droplet on PTFE at 25 °C at 0, 0.32, 0.6, and 1 ns in the XZ plane. Panels (**e**–**g**) illustrate interfacial area growth over time for 20, 25, and 30 nm droplets, with dashed lines indicating stabilization points.

The 12 cubic configurations in Table 1 were equilibrated, forming 20, 25, and 30 nm droplets. Figure 2 shows the equilibration of a 20 nm droplet (panels a–d) and interfacial area growth on PTFE for 20, 25, and 30 nm droplets (panels e–g). Initially, at 0 ns, droplets were compact and square, with no surface interaction. By 0.32 ns, they spread and transitioned to a spherical shape due to surface tension and adhesion. By 0.32 ns, the droplet flattened further against the surface, stabilizing between 0.6 ns and 1 ns into an equilibrium state with a consistent spherical cap and volume. The total energies are provided in the Supporting Information document stabilized after initial fluctuations, confirming equilibrium for each droplet size and condition, including temperature and salinity variations. At equilibrium, theoretical wetting methods were used to calculate the contact angle, with stable molecular dynamics data. The process was described by Blake and Haynes’ molecular-kinetic theory (MKT) [30]. Changing of the contact angle θ(t) was formulated using Equation (2) for a droplet characterized by a spherical cap of volume V:

(2) $$\frac{d\theta }{dt}=-{\left(\frac{\pi }{3V}\right)}^{1/3}\frac{{\left(2-3cos\theta +{cos}^{3}\theta \right)}^{4/3}}{(1-cos\theta {)}^{2}}\frac{dR}{dt}$$

where R is the droplet’s interfacial radius (S) as a spherical cap; the traditional MKT model assumes a uniform surface with consistent liquid–solid interactions.

**Figure 3:**
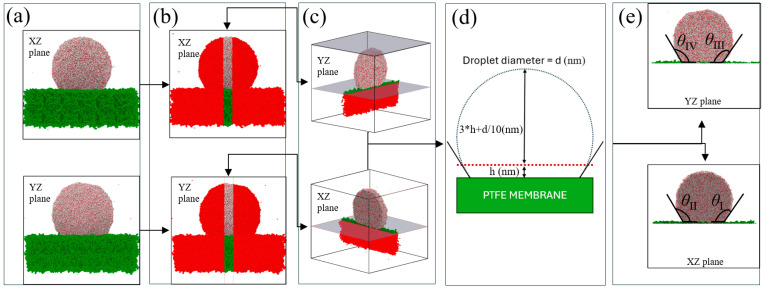
Methodology for 20 nm droplet contact angle measurement: (**a**) Equilibration at 25 °C in XZ and YZ views, (**b**) 30 Å central section removed, (**c**) cross-sections post-removal, (**d**) contact angle equation, (**e**) tangent-based angle determination with PTFE surface.

However, real surfaces often exhibit heterogeneity, such as chemical variations, fouling, or roughness, impacting wetting behavior [31]. Consequently, MKT’s limitations include neglecting surface roughness. Hautman and Klein [32] proposed Equation (3) linking the droplet’s center of mass ⟨z_com_⟩ to the contact angle, assuming constant droplet density and using the equation cos θ = (1 − h/r):

(3) $$\left\langle z_{\mathrm{com}}\right\rangle =2^{-4/3}R_{0}{\left(\frac{1-cos\theta }{2+cos\theta }\right)}^{1/3}\frac{3+cos\theta }{2+cos\theta }$$

where h is the height and r is the radius, and z_com_ is the droplet’s center of mass z-coordinate relative to the surface, with ⟨…⟩ as the time-averaged value. R_0_ = 3N/4πρ0 is the radius of the best-fit free sphere, where “N” is the total number of molecules and ρ0 (0.033 Å^−3^ for water) is the bulk liquid density. The z_com_ value was obtained from the equilibrated droplet mass center height values measured from the plot in the Supporting Information document for 20, 25, and 30 nm droplets at 25 °C. The z_com_ value was then inserted into Equation (3), resulting in contact angles of 132–135°, which are 11–14° higher than the experimental value reported by Yijie Xiang et al. [13]. New methods have been developed to improve contact angle estimation due to inaccuracies in MKT and center of mass approaches. Fan and Cagin [33] refined Hautman and Klein’s method by enhancing the center of mass calculations without assuming density uniformity. The height (h) and radius (r) of the best-fit partial sphere were used to directly calculate the contact angle as cos θ = (1 − h/r). Ruijter et al. [34] used droplet density profiles to find the liquid–gas interface, but this method could yield inconsistent results for small droplets and uneven surfaces [35]. Santiso et al. [36] proposed a surface meshing technique for local contact angle estimation, noting that accuracy depended on correctly identifying the contact layer. However, the accuracy of this method, like others relying on interface recognition, depended heavily on the density profile used to identify the contact layer. The study noted that a fine mesh could mistakenly classify density fluctuations within the droplet as part of the interface. Khalkhali et al. [37] used a convex hull algorithm to model the droplet surface with triangles, calculating angles between normals and the solid surface without assuming droplet shape or using a density profile. However, contact angle hysteresis and local variations on rough surfaces, which could create complex contact line geometries, were not fully captured by this method, potentially leading to missed nuances. After equilibrium in Figure 3a, 3 nm thick planes were defined perpendicular to the xz and yz planes, and areas outside the center planes, highlighted in red in Figure 3b, were removed. Root mean square roughness (R_q_) and average roughness (R_a_) measurements were then measured, defined in Equation (4):

(4) $$R_{q}=\sqrt{\frac{1}{N}\sum_{i=1}^{N}{\left(z_{i}-\bar{z}\right)}^{2}},\;R_{a}=\frac{1}{N}\sum_{i=1}^{N}\left|z_{i}-\bar{z}\right|$$

where N is the total number of surface points, z_i_ represents the height of the i-th point, and is the average height. After aligning the z-values of all droplet configurations to the same baseline, the data below this axis, highlighted as red in Figure 3c, were excluded. The rough PTFE surface was filtered to match the droplet’s spreading area, and roughness parameters R_q_ and R_a_ were calculated using the adjusted z-values, ensuring consistent roughness for contact angle calculations. Density contours for each droplet configuration were generated from Figure 3c by dividing the droplet space into bins (xz plane: Δx = 0.3 nm, Δz = 0.3 nm, Δy = 3 nm; yz plane: Δy = 0.3 nm, Δz = 0.3 nm, Δx = 3 nm).

Water molecule data identified the water/vapor boundary at 0.5 g/cm^3^ density [9]. Once the droplet’s water/vapor boundary, dashed line Figure 3d, was established, the vertical distance to the droplet apex was measured, remaining constant across simulations after 0.8 ns. Data points along the interface captured droplet curvature at the PTFE contact edges, with a circular arc fitted to estimate the slope at the contact points. Tangent lines defined by these slopes provided contact angles (θ_I_, θ_II_ on xz plane; θ_III_, θ_IV_ on yz plane), which were averaged as shown in Figure 3e.

## 3. Results and Discussion

**Figure 4:**
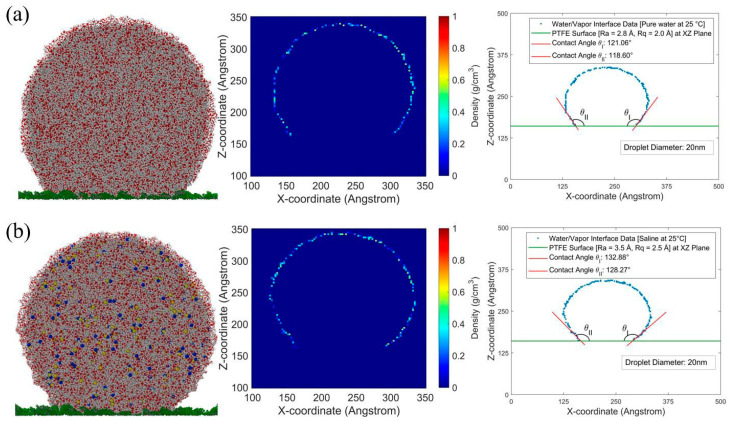
Effect of salinity on contact angles: 20 nm pure (**a**) and saline (**b**) water droplets, 3 nm thick, on a PTFE surface at 25 °C in the XZ plane, with density contours and angle measurements.

In Figure 4a,b, bins with densities above 0.5 g/cm^3^ [9] were excluded to isolate the water phase and define the water/vapor interface. The method from Figure 3d was applied to each droplet configuration to consistently measure the contact angle.

**Figure 5:**
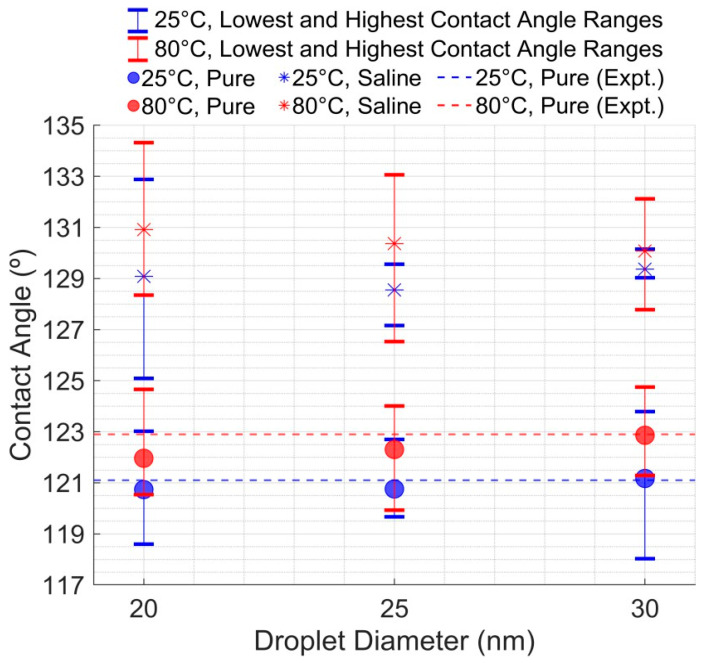
Effect of Droplet Diameter and Temperature on Contact Angle for Pure and Saline Solutions.

Two distinct effects were observed when plotting the contact angle values from equilibrated droplet configurations, including the lowest and highest contact angle ranges shown in Figure 5, and by comparing the results with experimental data for pure droplets at 25 °C and 80 °C [13]. Temperature Effect: A slight increase in contact angle with rising temperature was noted for both saline and pure droplets, showing higher angles at 80 °C compared to 25 °C, indicating increased surface wettability. Salinity Effect: Saline systems consistently showed higher contact angles than pure water across all droplet sizes (20, 25, and 30 nm), with an increase of 8–9 degrees, indicating that salt presence significantly altered droplet–surface interactions. The detailed measurements and 1 ns equilibrated visualizations for all droplet sizes are provided in the Supplementary Materials. The effects of salinity and temperature are discussed in the next section.

### Salinity and Temperature Effects

**Figure 6:**
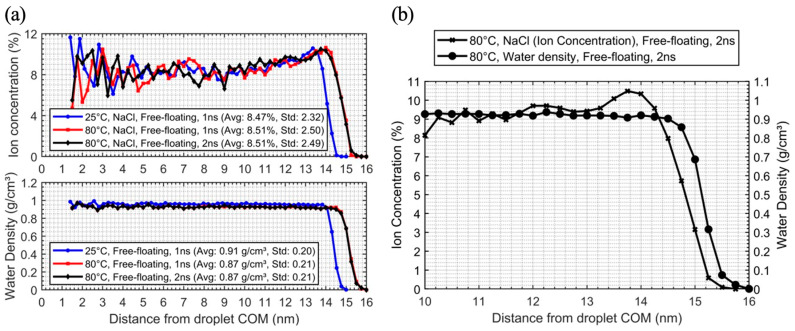
(**a**) Ion mass concentration, water density profiles, and (**b**) partial pressure of a NaCl nanodroplet at 25 °C and 80 °C over time (Avg = average intensity, Std = standard deviation).

**Figure 7:**
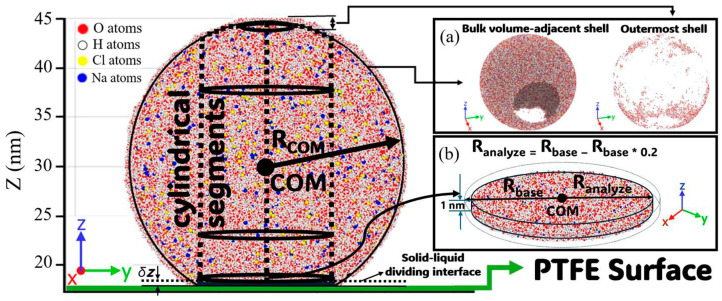
The analysis setup for a 30 nm droplet at 25 °C in the YZ plane, used to assess salinity and temperature effects. (**a**) Illustration of the boundary between the outermost and adjacent bulk shell. (**b**) Determination of the contact interface of the contact angle.

The impact of salinity and temperature on contact angle variation was studied using 30 nm saline and pure droplets on PTFE at 25 °C and 80 °C, as well as equilibrated free-floating droplets at these temperatures. The initial analysis divided the free-floating droplets into 0.25 nm spherical shells from 1.5 nm away from the COM outward, with the region within 1.5 nm defined as the bulk. Figure 6a,b presents a detailed analysis of a NaCl nanodroplet, showing ion mass concentration and water density at 25 °C and 80 °C over 1 ns and 2 ns timescales. Ion mass concentration in the free-floating droplets gradually migrated toward the surface, indicating surface segregation in Figure 7a,b. At 80 °C, the peak shifted outward compared to 25 °C, consistent with thermal expansion. The 80 °C, 2 ns simulation matched the 1 ns run, with ion concentration peaking at 13.8–14 nm before declining sharply, showing NaCl ions accumulated near the surface.

Water density remained constant until 13.8–14 nm, then dropped, marking the liquid–vapor transition and confirming surface segregation. This method was applied to 30 nm droplets at 25 °C on PTFE, but the droplet’s non-spherical shape in Figure 7a complicated direct comparisons with free-floating droplets. A different approach was applied due to this non-uniformity, so the cylindrical segments method was applied to determine z-profiles, as illustrated in Figure 7.

**Figure 8:**
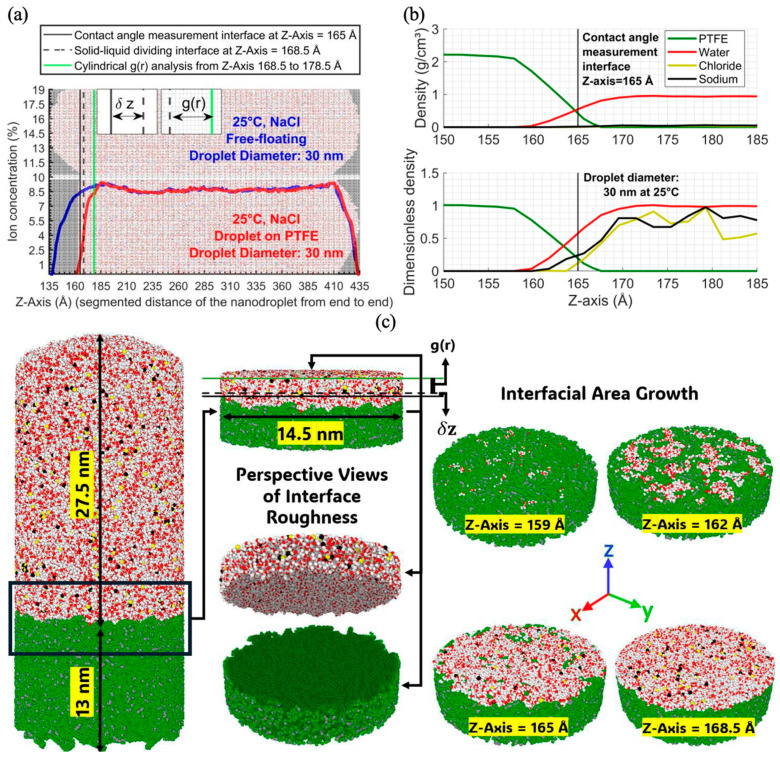
Ion mass concentration and density profiles of 30 nm NaCl nanodroplets: (**a**) Free- floating vs. PTFE-adsorbed droplets, (**b**) density and dimensionless density near the PTFE interface (normalized using reference densities of PTFE, water, Na, and Cl), and (**c**) interfacial roughness and spreading area growth visualization.

**Figure 9:**
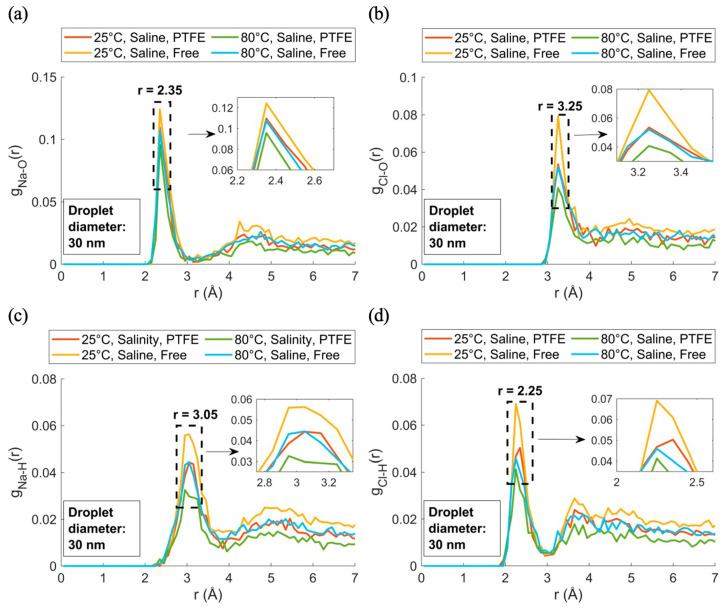
RDFs under various temperature and salinity conditions with/without a PTFE membrane: (**a**) Na-O, (**b**) Cl-O, (**c**) Na-H, (**d**) Cl-H.

The first center of mass (COM) was identified, and the R_analyze_ radius was determined based on the droplet’s R_base_ radius in Figure 7b. The PTFE and free-floating droplets were then segmented along the area defined by R_analyze_ and divided into 0.3 nm thick sections along the z-axis to analyze NaCl mass concentration and density distributions, Figure 8a. Ion concentration was about 1% below the contact angle interface, Figure 8b, rising to 4% at the solid–liquid dividing interface and reaching 8.5% at the top of the cylindrical analysis, showing significant ion segregation from the PTFE surface. Intermediate regions showed similar profiles for both free-floating and PTFE droplets, indicating surface interactions primarily affected ions near the PTFE–liquid interface. This region is magnified in Figure 8b, showing density and dimensionless density, and is visualized in Figure 8c for interface roughness and area growth. Discrepancies in ion distribution between free-floating and PTFE droplets were analyzed using RDF for 1 nm thick samples (168.5 Å to 178.5 Å), Figure 8c, excluding surface roughness effects. This range represents the spreading area determining the contact angle, with RDF results examining interactions between Na^+^, Cl^−^, and water, including temperature effects on spreading and contact angle. Figure 9 presents the RDF results for sodium–oxygen (g_Na-O_(r)), chloride–oxygen (g_Cl-O(_r)), sodium–hydrogen (g_Na-H_(r)), and chloride–hydrogen (g_Cl-H_(r)) interactions for saline nanodroplets at different temperatures with and without a PTFE membrane.

Peaks were compared with experimental data in Table 2, showing distinct ion-specific hydration structures influenced by temperature and salinity. The highest peak intensity was observed in the 25 °C, Saline, Free-floating condition. The 25 °C, Saline, PTFE, and 80 °C, Saline, Free-floating cases had equal intensities, while the 80 °C, Saline, PTFE condition had the lowest. This trend indicated stronger ion–water interactions in free-floating droplets at 25 °C and weaker interactions near PTFE, especially at 80 °C.

In Figure 9a, the primary peak g_Na-O_(r) at r = 2.35 Å corresponds to the first hydration shell of water around the sodium ion [39]. The lowest peak intensity was seen in the PTFE case, indicating weaker ion–water interactions near the PTFE surface at higher temperatures. Similar sodium ion hydration behavior was noted for 25 °C, Free, and 80 °C, PTFE, suggesting comparable disruption by temperature and membrane effects. Figure 9b shows g_Cl-O_(r) with a first peak at r = 3.25 Å. The 25 °C, Saline, Free-floating case had the highest peak intensity, while 25 °C, Saline, PTFE and 80 °C, Saline, Free-floating showed equal intensities. The 80 °C, Saline, PTFE condition had the lowest peak, indicating reduced ion–water structuring near the membrane at higher temperatures.

**Figure 10:**
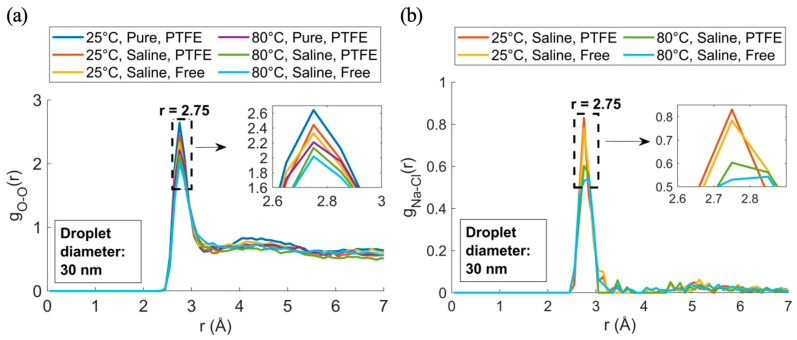
RDFs under various temperature and salinity conditions with/without a PTFE membrane: (**a**) O-O, (**b**) Na-Cl.

In Figure 9c, g_Na-H_(r) showed a peak at r = 3.05 Å, indicating that the hydrogen atoms were farther from the sodium ions compared to the oxygen atoms in g_Cl-O_(r). This suggested water molecules oriented their oxygen atoms toward Na^+^, forming hydrated ions. Figure 9d shows g_Cl-H_(r) with a first peak at r = 2.25 Å, lower than g_Cl-O_(r), indicating chloride ions interacted more strongly with hydrogen in free-floating droplets at lower temperatures, while PTFE surfaces and higher temperatures weakened these interactions. The g_O-O_(r) peak at 2.75 Å in Figure 10a matched the oxygen–oxygen RDF from X-ray diffraction of liquid water [40].

At 25 °C, the interfacial area for the pure case showed a higher g_O-O_(r) peak at r = 2.75 Å, indicating stronger water structuring. This caused moderate droplet expansion, less than at higher temperatures. In the 25 °C saline case, the interfacial area was smaller, consistent with the reduced g_O-O_(r) peak, as salt disrupted the water structure, limiting expansion and increasing the contact angle. At 80 °C, observations for both saline and pure cases followed this trend. Weaker water structuring (lower g_O-O_(r) peak) correlated with greater droplet spreading and larger interfacial area. Salinity further restricted expansion by disrupting the water network. Figure 10a shows Na–Cl interactions were more temperature-sensitive, with a stronger structure at lower temperatures, diminishing at higher temperatures, especially near the PTFE surface. g_O-O_(r) structuring: as water structuring weakened (as indicated by a lower g_O-O_(r) peak), the droplet spread more, resulting in a larger interfacial area. Salinity further reduced this expansion by disrupting the water network. In Figure 10b, Na–Cl interactions were shown to be more temperature sensitive, with a more evident structure at lower temperatures, which diminished at higher temperatures, especially near the PTFE surface.

## 4. Conclusions

In summary, this study demonstrated that salinity and temperature were factors in modulating the wettability of water droplets on hydrophobic PTFE surfaces. The addition of NaCl was found to increase contact angles by 6% across all droplet sizes (20, 25, and 30 nm). Elevated temperatures also led to increased contact angles, although their effect was less pronounced compared to that of salinity. The RDF analyses revealed ion segregation and reduced water structuring at the liquid–solid interface. The natural structuring of water was significantly disrupted by the presence of salt, primarily due to the weakening of its hydrogen bonding network, which limited the droplet’s ability to spread over the PTFE surface. This reduction in spreading resulted in a smaller interfacial area and, consequently, a higher contact angle. While elevated temperatures contributed to some degree of disruption by promoting expansion and increasing the interfacial area, the effect of salinity on water structuring was more pronounced. Thus, the salt-induced disruption was the dominant factor in reducing water structuring and increasing the contact angle, with temperature having a secondary but notable impact on the behavior of the droplet. These findings can be used to design optimal hydrophobic surfaces for the distillation process based on the working temperature and salt conditions. The hydrophobicity of the membrane surface is enhanced by increased salinity and temperature, which influences salt rejection rates in the membrane distillation process. As hydrophobicity increases, water wettability decreases, preventing liquid water from penetrating membrane pores and ensuring vapor-only transport. This effect improves salt rejection efficiency by minimizing the risk of pore wetting.

## Figures and Tables

**Table 1:** The configuration details of six different cubic saline cases.

Configurations	N_water_	N_Na+_	N_Cl−_	C_NaCl_
16 (equivalent to 20 nm sphere)	130,600	3700	3700	8.45%
20 (equivalent to 25 nm sphere)	253,546	7227	7227	8.45%
24 (equivalent to 30 nm sphere)	440,024	12,488	12,488	8.45%

**Table 2:** Comparison of first peak positions in RDFs of experimental NaCl solutions (with standard deviations) and MD simulations from this study.

	Methods		
RDF Pairs	X-Ray Spectra	Neutron Diffraction	MD (This Study)
Na-O	2.35	2.34 (0.14)	2.35
Na-H	-----	2.97 (0.12)	3.05 (0.15)
Cl-O	3.20	3.16 (0.11)	3.25
Cl-H	-----	2.19 (0.16)	2.25 (0.10)

X-ray spectra peaks (QMax = 24 Å^−1^) were obtained from reference [38], and neutron diffraction peaks (QMax = 16 Å^−1^) from reference [39].

## Data Availability

The original contributions presented in the study are included in the article, and further inquiries can be directed to the corresponding author.

## Supplementary Materials

The following supporting information can be downloaded at: https://www.mdpi.com/article/10.3390/membranes15020058/s1.
