# Supplementary material for: Hydrophobic Membrane Wettability: Effects of Salinity and Temperature
Source: Membranes (Basel). 2025 Feb 9;15(2):58. doi: 10.3390/membranes15020058 (PMC11857761; doi:10.3390/membranes15020058)

Simulation Setting and Molecular Interactions

The simulations used real units, full atom style, and 3D periodic boundaries. Atom interactions in the following equation were modeled with Lennard–Jones and Coulombic potentials (cutoff: 10 Å) and the lj/cut/coul/long pair style, with long-range electrostatics calculated by the PPPM method [1] at 10^{-5} precision and fourth-order k-space accuracy:

$$u_{ij}(r) = 4\varepsilon_{ij}\left[\left(\frac{\sigma_{ij}}{r}\right)^{12} - \left(\frac{\sigma_{ij}}{r}\right)^{6}\right] + \frac{1}{4\pi\varepsilon_0}\frac{q_i q_j}{r}$$

where r indicates the distance between positions i and j; q_i and q_j refer to the electric charge at sites i and j, respectively. The ε_0 is the vacuum permittivity, ε_{ij} represents the well depth of the LJ potential, and σ_{ij} is the characteristic diameter. The mixed pair coefficients were generated between atoms of type i and j using the arithmetic mixing combination rule: $\epsilon_{ij}=\sqrt{\epsilon_i\epsilon_j}$, and $\sigma_{ij}=(\sigma_i+\sigma_j)/2$. The system was composed of F (fluorine), C (carbon) in CF_3 groups, C in CF_2 groups for PTFE, H_2O (water), Na^+ (sodium ions), and Cl^- (chloride ions). PTFE parameters were sourced from [2–3]. The SPC/E (extended) water model [4] was used to establish a nanoscale bulk water system. The OPLS-derived Lennard–Jones and charge parameters for the Na^+ and Cl^- were obtained from the GROMACS software [5]. Non-bonded parameters are in the following Table.

Table. Lennard–Jones parameters and partial charges for PTFE, water, and ions.

Pair Coeffs	σ(nm)	ε (kJ/mol)	q(e)
F (PTFE)	2.5378	0.0634	-0.11018
CF_3 (PTFE)	2.8062	0.0838	0.33040
CF_2 (PTFE)	2.8812	0.0781	0.22050
O (water)	3.1655	0.1554	-0.84760
H (water)	0.0	0.0	0.42380
Na^+ (ion)	3.3304	0.0027	1
Cl^- (ion)	4.4172	0.1177	-1

The system was stabilized using the Nosé–Hoover thermostat [6] at 298.15 K and 353.15 K, with individual group control for accurate thermal simulation. The velocity-Verlet algorithm integrated velocities and positions with a 2.0 fs time step.

References

1. Darden, T.; York, D.; Pedersen, L. Particle Mesh Ewald: An N ·log(N) Method for Ewald Sums in Large Systems. J. Chem. Phys. **1993**, 98 (12), 10089–10092. https://doi.org/10.1063/1.464397.
2. Kaya, O.; Oztekin, A.; Webb, E. B. Development of AMBER-Compliant Transferable Force Field Parameters for Polytetrafluoroethylene. Open Chem. **2024**, 22 (1), 20240072. https://doi.org/10.1515/chem-2024-0072.
3. Kaya, O. Data from "Development of an AMBER-Compliant Transferable Force Field Parameters for Polytetrafluoroethylene," **2024**, 322406 Bytes. https://doi.org/10.6084/M9.FIGSHARE.26197325.V1
4. Berendsen, H. J. C.; Grigera, J. R.; Straatsma, T. P. The Missing Term in Effective Pair Potentials. J. Phys. Chem. **1987**, 91 (24), 6269–6271. https://doi.org/10.1021/j100308a038.
5. Abraham, M. J.; Murtola, T.; Schulz, R.; Páll, S.; Smith, J. C.; Hess, B.; Lindahl, E. GROMACS: High Performance Molecular Simulations through Multi-Level Parallelism from Laptops to Supercomputers. SoftwareX **2015**, 1–2, 19–25. https://doi.org/10.1016/j.softx.2015.06.001
6. Nosé, S. A Molecular Dynamics Method for Simulations in the Canonical Ensemble. Mol. Phys. **1984**, 52 (2), 255–268. https://doi.org/10.1080/00268978400101201.

Total energies for (a) 30 nm, (b) 25 nm, and (c) 20 nm droplets over simulation time.

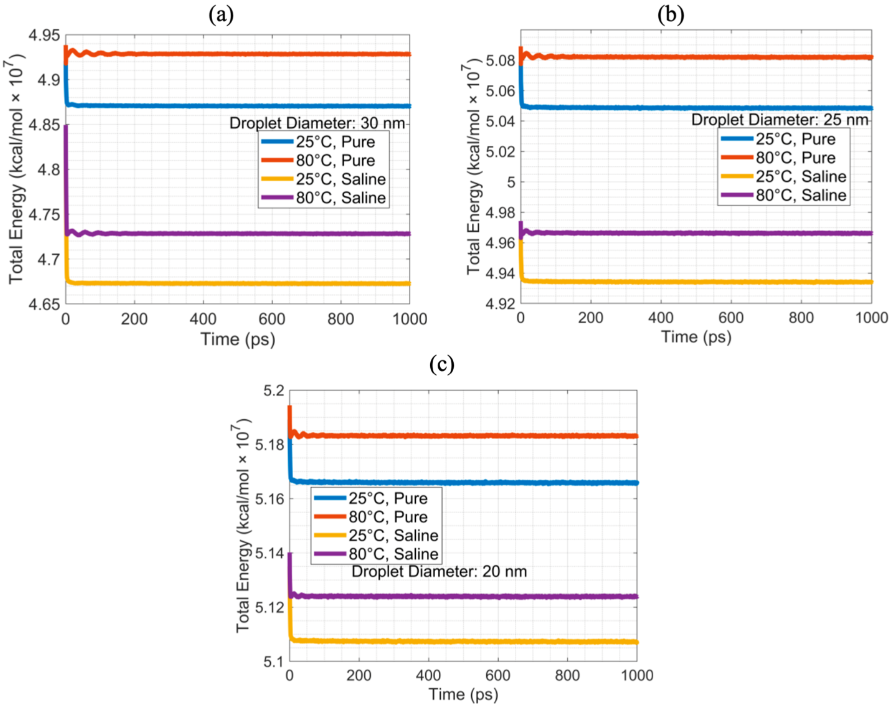

Effect of temperature and salinity on droplet mass center height for (a) 30 nm, (b) 25 nm, and (c) 20 nm droplets on a PTFE surface.

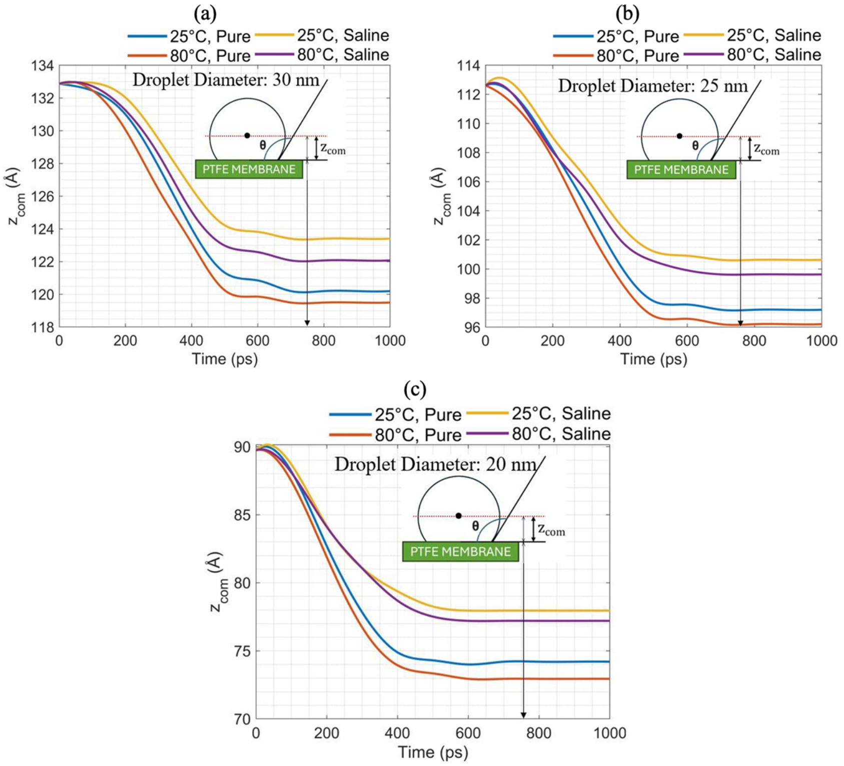

Supplement: Supplementary file 1 [file membranes-15-00058-s001.zip › membranes-3415435-supplementary.pdf]
